# Experiences and strategies adopted for the implementation of pharmaceutical services in hospital geriatric units: A scoping review protocol

**DOI:** 10.1016/j.rcsop.2023.100262

**Published:** 2023-04-05

**Authors:** Alan Maicon de Oliveira, Fabiana Rossi Varallo, Leonardo Régis Leira Pereira

**Affiliations:** School of Pharmaceutical Sciences of Ribeirão Preto, University of São Paulo, Ribeirão Preto, São Paulo, Brazil

**Keywords:** Geriatrics, Patient care team, Patient-centered care, Patient safety, Pharmaceutical services, Review

## Abstract

**Background:**

Pharmaceutical care is considered an important pillar for promoting the rational and safe use of medicines. Consequently, it constitutes actions of practices capable of reducing morbidity and mortality induced by pharmacotherapy. On the other hand, pharmaceutical services may face several barriers related to the implementation of these practices. These difficulties are associated with management, availability of an appropriate physical environment, integration with the multidisciplinary team, and acceptance of pharmaceutical interventions by health professionals.

**Objectives:**

This study aims to map and summarize the scientific evidence on the experiences and strategies used to implement pharmaceutical services in hospital geriatric units.

**Methods:**

The scoping review will be based on three electronic databases (PubMed, EMBASE, and Web of Science). Studies that met the inclusion criteria and are published by December 2022 will be selected. The screening, eligibility, extraction, and assessment of studies will be carried out by two independent researchers. Experimental and observational studies will be eligible for inclusion.

**Discussion:**

The experiences of incorporating pharmaceutical care into geriatric hospital units need to be better disseminated. Our review could support the performance of pharmaceutical care in other geriatric wards and has the potential to be a reference for multidisciplinary training. In addition, the study is related to the global challenge of the World Alliance for Patient Safety as it is a survey that will demonstrate strategies for safety in the use of medicines.

## 1 Introduction

The irrational use of medicines is a major public health problem, causing up to 15% of hospitalizations.^1^ This problem becomes more worrying with the practice of polypharmacy, that is, the excessive and inappropriate use of medication.^2^ Especially when this event occurs in older people, who have physiological changes inherent to the aging process, which favor changes in the pharmacokinetics and pharmacodynamics of drugs.^3^

Multiple comorbidities generally affect older people, which generates the search for treatments in different health services and medical specialties. The lack of integration in the transition of care and communication between health professionals is associated with inadequate prescriptions, unnecessary polypharmacy, the occurrence of adverse events, increased drug interactions, and poor adherence to treatment.^4^ To improve this situation, it is proposed that pharmaceutical care contributes to the reduction of these problems.^5^

Pharmaceutical care is the set of actions and services carried out by the pharmaceutical professional, taking into account the concepts of the individual, family, community, and health team, with a focus on the prevention and resolution of health problems, in addition to their promotion, protection, prevention of damage and recovery, including not only the clinical-assistance dimension but also the technical-pedagogical dimension of health work.^6^

Pharmaceutical care is based on person-centered care, meeting the population's health needs and seeking to provide autonomy, as well as making the individual part of their treatment, adapting therapeutic goals according to existing possibilities and reality.^5^^,^^7^

Pharmaceutical services performed in the context of pharmaceutical care improve the clinical outcomes of individuals, increase the quality of life, and reduce hospitalization rates, use of health services, and medical expenses.^8^

Considering what was previously described, pharmaceutical care is considered an important pillar for promoting the rational and safe use of medicines. Consequently, it constitutes actions of practices capable of reducing morbidity and mortality induced by pharmacotherapy, especially in the elderly population.^9^

On the other hand, pharmaceutical services may face several barriers related to the implementation of these practices and also depending on the health area in which they will be inserted. These difficulties are associated with management, availability of an appropriate physical environment, integration with the multidisciplinary team, and acceptance of pharmaceutical interventions by health professionals, in addition to other problems that involve the consolidation of practices in health care.^10^^,^^11^ In addition, the experiences of incorporating pharmaceutical care into geriatric hospital units need to be better disseminated.^12^

Therefore, the aims of our scope review will be to map and summarize the scientific evidence on the experiences and strategies used to implement pharmaceutical services in hospital geriatric units.

## 2 Methods

### 2.1 Design of the protocol

We will conduct our scoping review based on the methodology developed by Joanna Briggs Institute (JBI),^13^ and in line with the Preferred Reporting Items for Systematic Reviews and Meta-Analyses extension for scoping reviews (PRISMA-ScR) checklist.^14^ This method will be used to answer the following guiding question: What are the lived experiences and strategies used for implementation and which pharmaceutical services are implemented in geriatric wards?

### 2.2 Eligibility criteria

The inclusion and exclusion criteria of the studies (See Table 1) will be related to the guiding question, which was elaborated according to the acronym PCC (Population, Concept and Context).^13^ Therefore, the inclusion criteria will include:

▪ *Population:* Geriatric hospital wards.
▪ *Concept:* Studies that highlight the experiences and strategies used to implement pharmaceutical services.
▪ *Context:* Implementation of pharmaceutical services and description of services developed (technical and clinical).

**Table 1:** Inclusion and exclusion criteria for studies.

INCLUSION	EXCLUSION
Studies describing the experiences and strategies for implementing pharmaceutical care in geriatric hospital units.	Studies describing the experiences and strategies for implementing/conducting pharmaceutical care in other health units.
Experimental and observational studies.	Review studies, guidelines and organizational recommendations, protocol studies, editorials, lectures, editor letters, books, book chapters, and/or abstracts presented at conferences.
Written Studies in English, Spanish, and Portuguese.	Written Studies in other languages.

In addition, studies published by the end date of December 31, 2022, will be considered. Only studies written in English, Spanish, and Portuguese will be considered during the stages of screening.

Publications will be excluded if they are: review studies, guidelines and organizational recommendations, protocol studies, editorials, lectures, letters to the editor, books, book chapters, and/or abstracts presented at conference venues.

### 2.3 Search strategy

The search will be performed, using Medical Subject Headings (MeSH), and according to a predefined strategy (Supplementary material 1) in the following databases: PubMed, EMBASE, and Web of Science. Our search strategy was also validated by our University librarian.

### 2.4 Selection of sources of evidence and data collection process

**Fig. 1:**
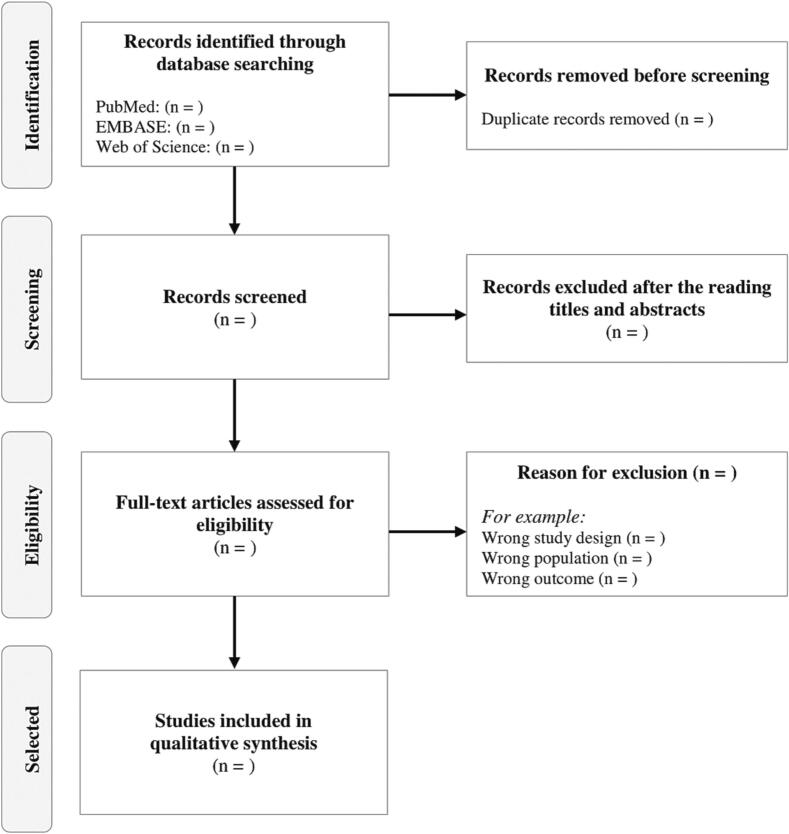
Flowchart of study selection and inclusion process.

Rayyan QCRI^15^ will be used and all references found will be inserted into this software, in addition, duplicate studies will be excluded. After that, two independent reviewers will analyze the titles and abstracts of the studies found. If there are disagreements, a third reviewer will be consulted to resolve them. Then, the reviewers will evaluate the eligibility of the studies according to the reading of the full text (Fig. 1).

A Microsoft Office Excel® spreadsheet will be used for data extraction. The following variables will be extracted:

▪ Structural characteristics of the studies (authors, year of publication, country of origin, type of study, and aims);
▪ Characteristics of the geriatric wards where the studies were carried out (type of hospital, number of beds, estimated number of patients, and professionals working in the multidisciplinary team);
▪ Characteristics of implemented pharmaceutical services (type of service, year of implementation, the method used, number of pharmaceutical professionals working in the services, duration of work activities per day, number of patients attended by pharmaceutical services, indicators (clinical, humanistic, and/or economic) evaluated);
▪ Qualitative outcomes related to the experiences and strategies used in the implementation of pharmaceutical services (for example, practice experience, barriers, facilitators, improvements, learnings, strengths or weaknesses).

Some changes can be made in the data extraction process according to the needs identified during the study.

### 2.5 Synthesis of results

The systematization of the extracted data will be developed by the transcription of the data in tables and figures.

### 2.6 Protocol registration

The development protocol for our scoping review was registered on the Open Science Framework (OSF) portal and is available at: <10.17605/OSF.IO/JCVFX www.doi.org/10.17605/OSF.IO/JCVFX>.

## 3 Discussion

Our study will be dedicated to identifying experiences and strategies used to implement pharmaceutical care in hospital geriatric wards. In January 2023, an advanced search was carried out in the OSF study protocol registry database (www.osf.io/registries), and no study was found with an objective and method similar to the one we are proposing for the topic of our scoping review. In this way, the originality related to our proposal is suggested.

The importance of our study is based on scientific evidence that reinforces the beneficial and satisfactory impacts arising from health care with shared decision-making, where the integration of pharmacists, a multidisciplinary team, and the users of geriatric units themselves stands out. These impacts highlight reductions in hospitalization rates, problems related to pharmacotherapy and bed occupancy, as well as increased patient safety and satisfaction.^4^^,^^8^^,^^10^^,^^16^

Our review could support the performance of pharmaceutical care in other geriatric wards and has the potential to be a reference for multidisciplinary training. In addition, the study is related to the global challenge of the World Alliance for Patient Safety, which is coordinated by the World Health Organization and encourages research to develop strategies for safety in the use of medicines.

## Funding

This study was financed in part by the Coordenação de Aperfeiçoamento de Pessoal de Nível Superior - Brasil (CAPES) [Finance Code: 88882.328412/2019-01].

## Declaration of Competing Interest

The authors have no relevant financial or non-financial interests to disclose.

## Appendix A Supplementary data

Supplementary material Image 1
