# Supplementary material for: Experiences and strategies adopted for the implementation of pharmaceutical services in hospital geriatric units: A scoping review protocol
Source: Explor Res Clin Soc Pharm. 2023 Apr 5;10:100262. doi: 10.1016/j.rcsop.2023.100262 (PMC10172926; doi:10.1016/j.rcsop.2023.100262)
Supplement: Supplementary file 1 — Supplementary material [file mmc1.pdf]

Supplementary Information

Supplementary material 1 - Search strategy adapted to databases

1. PUBMED

	**Search strategy**
**#1**	("Ward*"[tiab] OR "Infirmar*"[tiab] OR "Patients' Rooms"[mesh] OR "Patients' Room*"[tiab] OR "Patients Room*"[tiab] OR "Patient Room*"[tiab] OR "Patient's Room*"[tiab] OR "Private Room*"[tiab] OR "Semi-Private Room*"[tiab] OR "Semi Private Room*"[tiab]) OR ("Hospitals"[mesh] OR "Hospital*"[tiab] OR "Tertiary Care Centers"[mesh] OR "Tertiary Care Center*"[tiab] OR "Tertiary Referral Center*"[tiab] OR "Tertiary Referral Hospital*"[tiab] OR "Tertiary Hospital*"[tiab]) AND ("Geriatrics"[mesh] OR "Geriatrics"[tiab] OR "Gerontology"[tiab] OR "Health Services for the Aged"[mesh] OR "Geriatric Health Service*"[tiab] OR "Health Services for the Elderly"[tiab] OR "Health Services for Aged"[tiab] OR "Aged"[mesh] OR "Aged"[tiab] OR "Elderly*"[tiab] OR "Senior*"[tiab] OR "Old-aged"[tiab] OR "Older people"[tiab] OR "Older person"[tiab] OR "Older Individual*"[tiab] OR "Aged, 80 and over"[mesh])
**#2**	("Pharmaceutical care*"[tiab] OR "Pharmaceutical Services"[mesh] OR "Pharmaceutical Service*"[tiab] OR "Pharmaceutic Service*"[tiab] OR "Pharmacy Service*"[tiab] OR "Pharmacy Service, Hospital"[mesh] OR "Hospital Pharmacy Service*"[tiab] OR “Hospital Pharmaceutical Service*”[tiab] OR "Clinical Pharmacy Service*"[tiab] OR "Clinical Pharmacy"[tiab] OR "Evidence-Based Pharmacy Practice"[mesh] OR "Evidence-Based Pharmacy Practice*"[tiab] OR "Evidence Based Pharmacy Practice*"[tiab] OR "Evidence-Based Pharmaceutical Care*"[tiab] OR "Evidence Based Pharmaceutical Care*"[tiab] OR "Evidence-Based Pharmac*"[tiab] OR "Evidence Based Pharmac*"[tiab] OR "Pharmacy Practice*"[tiab]) AND ("Organizational Innovation"[mesh] OR "Organizational Innovation*"[tiab] OR "Organizational Change*"[tiab] OR "Implementation Science"[mesh] OR "Implementation Science*"[tiab] OR "Implement*"[tiab] OR "Develop*"[tiab] OR "Execut*"[tiab] OR "Appl*"[tiab] OR "Perform*"[tiab] OR "Realiz*"[tiab] OR "Accomplish*"[tiab] OR "Innovat*"[tiab] OR "Deploy*"[tiab] OR "Post-deploy*"[tiab] OR "Introduc*"[tiab] OR "Adopt*"[tiab] OR "Integrat*"[tiab] OR "Opening*"[tiab] OR "Origin*"[tiab] OR "Begin*"[tiab] OR "Start*"[tiab] OR "Arise"[tiab] OR "Arose"[tiab])
**#3**	("Experience*"[tiab] OR "Methods"[mesh] OR "Method*"[tiab] OR "Methodolog*"[tiab] OR "Strateg*"[tiab] OR "Instrument*"[tiab] OR "Tool*"[tiab] OR "Barrier*"[tiab] OR "Facilitator*"[tiab] OR "Health Services Accessibility"[mesh] OR "Access*"[tiab] OR "Motivation*"[mesh] OR "Expectation*"[tiab] OR "Incentive*"[tiab] OR "Disincentive*"[tiab] OR "Acceptance*"[tiab] OR "Disapproval*"[tiab] OR "Outcome Assessment, Health Care"[mesh] OR "Assess*"[tiab] OR "Outcome*"[tiab] OR "Procedure*"[tiab] OR "Program Evaluation"[mesh] OR "Evaluat*"[tiab] OR "Effectiveness"[tiab] OR "Solution*"[tiab] OR "Needs Assessment"[mesh] OR "Health Services Needs and Demand"[mesh] OR "Need*"[tiab] OR "Health Services Research"[mesh] OR "Process Assessment, Health Care"[mesh] OR "Process"[tiab] OR "Knowledge"[mesh] OR "Knowledge Discovery"[mesh] OR "Discover*"[tiab] OR "Knowledge"[tiab] OR "Management*"[tiab] OR "Systems Analysis"[mesh] OR "Cost-Benefit Analysis"[mesh] OR "Analysis"[tiab] OR "Monitoring"[tiab] OR "Quality"[tiab] OR "Guideline*"[tiab] OR "Patient Reported Outcome Measures"[mesh] OR "Health Knowledge, Attitudes, Practice"[mesh] OR "Attitude"[mesh] OR "Attitude*"[tiab] OR "Attitude of Health Personnel"[mesh] OR "Professional Practice"[mesh] OR "Institutional Practice"[mesh] OR "Practice Patterns, Pharmacists'"[mesh] OR "Evidence-Based Practice"[mesh] OR "Practice*"[tiab] OR "Organization and Administration"[mesh] OR "Administrati*"[tiab] OR "Administer*"[tiab] OR "Procedures and Techniques Utilization"[mesh] OR "Planning Techniques"[mesh] OR "Technique*"[tiab] OR "Planning"[tiab] OR "Evaluation Studies as Topic"[mesh] OR "Structuring"[tiab] OR "Patient Care Management"[mesh] OR "System*"[tiab] OR "Change Management"[mesh] OR "Change*"[tiab] OR "Benefit*"[tiab] OR "Risk"[mesh] OR "Risk*"[tiab] OR "Improvement*"[tiab] OR "Quality Indicators, Health Care"[mesh] OR "Indicator*"[tiab] OR "Health Care Quality, Access, and Evaluation"[mesh] OR "Quality of Health Care"[mesh] OR "Management Audit"[mesh] OR "Audit"[tiab] OR "Guideline Adherence"[mesh] OR "Adherence"[tiab] OR "Expert Testimony"[mesh] OR "Public Opinion"[mesh] OR "Opinion*"[tiab] OR "Learning"[mesh] OR "Learning"[tiab] OR "Education, Professional"[mesh] OR "Fragility"[tiab] OR "Evolution*"[tiab] OR "Transformation*"[tiab] OR "Strength*"[tiab] OR "Weaknesse*"[tiab] OR "Recommendation*"[tiab] OR "Growth*"[tiab] OR "Research Report"[mesh] OR "Progress"[tiab] OR "Instruction*"[tiab] OR "Goals"[mesh] OR "Goal*"[tiab] OR "Organizational Objectives"[mesh] OR "Objective*"[tiab] OR "Advance*"[tiab] OR "Conflict*"[tiab] OR "Feedback"[mesh] OR "Feedback*"[tiab] OR "Behavior"[mesh] OR "Behavior*"[tiab] OR "Capacity Building"[mesh] OR "Inservice Training"[mesh] OR "Training*"[tiab] OR "Qualification*"[tiab] OR "Professional Competence"[mesh] OR "Clinical Competence"[mesh] OR "Competence*"[tiab] OR "Aptitude"[mesh] OR "Ability*"[tiab] OR “Skill*”[tiab] OR "News"[tiab] OR "Preliminary Data"[mesh] OR "Culture"[mesh] OR "Culture*"[tiab] OR "Background*"[tiab] OR "Staff Development"[mesh] OR "Acceptability"[tiab]")

2. EMBASE

	**Search strategy**
**#1**	(“Ward*”:ab,ti OR “Infirmar*”:ab,ti OR “Patients Rooms":kw OR "Patients Room*":ab,ti OR "Patient Room*":ab,ti OR "Private Room*":ab,ti OR "Semi-Private Room*":ab,ti OR "Semi Private Room*":ab,ti) OR ("Hospitals":kw OR "Hospital*":ab,ti OR "Tertiary Care Centers":kw OR "Tertiary Care Center*":ab,ti OR "Tertiary Referral Center*":ab,ti OR "Tertiary Referral Hospital*":ab,ti OR "Tertiary Hospital*":ab,ti) AND ("Geriatrics":kw OR "Geriatrics":ab,ti OR "Gerontology":ab,ti OR "Health Services for the Aged":kw OR "Geriatric Health Service*":ab,ti OR "Health Services for the Elderly":ab,ti OR "Health Services for Aged":ab,ti OR "Aged":kw OR "Aged":ab,ti OR "Elderly*":ab,ti OR "Senior*":ab,ti OR "Old-aged":ab,ti OR "Older people":ab,ti OR "Older person":ab,ti OR "Older Individual*":ab,ti OR "Aged, 80 and over":kw)
**#2**	("Pharmaceutical care*":ab,ti OR "Pharmaceutical Services":kw OR "Pharmaceutical Service*":ab,ti OR "Pharmaceutic Service*":ab,ti OR "Pharmacy Service*":ab,ti OR "Pharmacy Service, Hospital":kw OR "Hospital Pharmacy Service*":ab,ti OR “Hospital Pharmaceutical Service*”:ab,ti OR "Clinical Pharmacy Service*":ab,ti OR "Clinical Pharmacy":ab,ti OR "Evidence-Based Pharmacy Practice":kw OR "Evidence-Based Pharmacy Practice*":ab,ti OR "Evidence Based Pharmacy Practice*":ab,ti OR "Evidence-Based Pharmaceutical Care*":ab,ti OR "Evidence Based Pharmaceutical Care*":ab,ti OR "Evidence-Based Pharmac*":ab,ti OR "Evidence Based Pharmac*":ab,ti OR "Pharmacy Practice*":ab,ti) AND ("Organizational Innovation":kw OR "Organizational Innovation*":ab,ti OR "Organizational Change*":ab,ti OR "Implementation Science":kw OR "Implementation Science*":ab,ti OR "Implement*":ab,ti OR "Develop*":ab,ti OR "Execut*":ab,ti OR "Appl*":ab,ti OR "Perform*":ab,ti OR "Realiz*":ab,ti OR "Accomplish*":ab,ti OR "Innovat*":ab,ti OR "Deploy*":ab,ti OR "Post-deploy*":ab,ti OR "Introduc*":ab,ti OR "Adopt*":ab,ti OR "Integrat*":ab,ti OR "Opening*":ab,ti OR "Origin*":ab,ti OR "Begin*":ab,ti OR "Start*":ab,ti OR "Arise":ab,ti OR "Arose":ab,ti)
**#3**	("Experience*":ab,ti OR "Methods":kw OR "Method*":ab,ti OR "Methodolog*":ab,ti OR "Strateg*":ab,ti OR "Instrument*":ab,ti OR "Tool*":ab,ti OR "Barrier*":ab,ti OR "Facilitator*":ab,ti OR "Health Services Accessibility":kw OR "Access*":ab,ti OR "Motivation*":kw OR "Expectation*":ab,ti OR "Incentive*":ab,ti OR "Disincentive*":ab,ti OR "Acceptance*":ab,ti OR "Disapproval*":ab,ti OR "Outcome Assessment, Health Care":kw OR "Assess*":ab,ti OR "Outcome*":ab,ti OR "Procedure*":ab,ti OR "Program Evaluation":kw OR "Evaluat*":ab,ti OR "Effectiveness":ab,ti OR "Solution*":ab,ti OR "Needs Assessment":kw OR "Health Services Needs and Demand":kw OR "Need*":ab,ti OR "Health Services Research":kw OR "Process Assessment, Health Care":kw OR "Process":ab,ti OR "Knowledge":kw OR "Knowledge Discovery":kw OR "Discover*":ab,ti OR "Knowledge":ab,ti OR "Management*":ab,ti OR "Systems Analysis":kw OR "Cost-Benefit Analysis":kw OR "Analysis":ab,ti OR "Monitoring":ab,ti OR "Quality":ab,ti OR "Guideline*":ab,ti OR "Patient Reported Outcome Measures":kw OR "Health Knowledge, Attitudes, Practice":kw OR "Attitude":kw OR "Attitude*":ab,ti OR "Attitude of Health Personnel":kw OR "Professional Practice":kw OR "Institutional Practice":kw OR "Practice Patterns, Pharmacists":kw OR "Evidence-Based Practice":kw OR "Practice*":ab,ti OR "Organization and Administration":kw OR "Administrati*":ab,ti OR "Administer*":ab,ti OR "Procedures and Techniques Utilization":kw OR "Planning Techniques":kw OR "Technique*":ab,ti OR "Planning":ab,ti OR "Evaluation Studies as Topic":kw OR "Structuring":ab,ti OR "Patient Care Management":kw OR "System*":ab,ti OR "Change Management":kw OR "Change*":ab,ti OR "Benefit*":ab,ti OR "Risk":kw OR "Risk*":ab,ti OR "Improvement*":ab,ti OR "Quality Indicators, Health Care":kw OR "Indicator*":ab,ti OR "Health Care Quality, Access, and Evaluation":kw OR "Quality of Health Care":kw OR "Management Audit":kw OR "Audit":ab,ti OR "Guideline Adherence":kw OR "Adherence":ab,ti OR "Expert Testimony":kw OR "Public Opinion":kw OR "Opinion*":ab,ti OR "Learning":kw OR "Learning":ab,ti OR "Education, Professional":kw OR "Fragility":ab,ti OR "Evolution*":ab,ti OR "Transformation*":ab,ti OR "Strength*":ab,ti OR "Weaknesse*":ab,ti OR "Recommendation*":ab,ti OR "Growth*":ab,ti OR "Research Report":kw OR "Progress":ab,ti OR "Instruction*":ab,ti OR "Goals":kw OR "Goal*":ab,ti OR "Organizational Objectives":kw OR "Objective*":ab,ti OR "Advance*":ab,ti OR "Conflict*":ab,ti OR "Feedback":kw OR "Feedback*":ab,ti OR "Behavior":kw OR "Behavior*":ab,ti OR "Capacity Building":kw OR "Inservice Training":kw OR "Training*":ab,ti OR "Qualification*":ab,ti OR "Professional Competence":kw OR "Clinical Competence":kw OR "Competence*":ab,ti OR "Aptitude":kw OR "Ability*":ab,ti OR “Skill*”:ab,ti OR "News":ab,ti OR "Preliminary Data":kw OR "Culture":kw OR "Culture*":ab,ti OR "Background*":ab,ti OR "Staff Development":kw OR "Acceptability":ab,ti)

3. Web of Science

	Search strategy
**#1**	TS=("Ward*" OR "Infirmar*" OR "Patients Rooms" OR "Patients Room*" OR "Patient Room*" OR "Private Room*" OR "Semi-Private Room*" OR "Semi Private Room*") **OR** ("Hospitals" OR "Hospital*" OR "Tertiary Care Centers" OR "Tertiary Care Center*" OR "Tertiary Referral Center*" OR "Tertiary Referral Hospital*" OR "Tertiary Hospital*") **AND** ("Geriatrics" OR "Geriatrics" OR "Gerontology" OR "Health Services for the Aged" OR "Geriatric Health Service*" OR "Health Services for the Elderly" OR "Health Services for Aged" OR "Aged" OR "Aged" OR "Elderly*" OR "Senior*" OR "Old-aged" OR "Older people" OR "Older person" OR "Older Individual*" OR "Aged, 80 and over")
**#2**	TS=("Pharmaceutical care*" OR "Pharmaceutical Services" OR "Pharmaceutical Service*" OR "Pharmaceutic Service*" OR "Pharmacy Service*" OR "Pharmacy Service, Hospital" OR "Hospital Pharmacy Service*" OR "Hospital Pharmaceutical Service*" OR "Clinical Pharmacy Service*" OR "Clinical Pharmacy" OR "Evidence-Based Pharmacy Practice" OR "Evidence-Based Pharmacy Practice*" OR "Evidence Based Pharmacy Practice*" OR "Evidence-Based Pharmaceutical Care*" OR "Evidence Based Pharmaceutical Care*" OR "Evidence-Based Pharmac*" OR "Evidence Based Pharmac*" OR "Pharmacy Practice*") AND ("Organizational Innovation" OR "Organizational Innovation*" OR "Organizational Change*" OR "Implementation Science" OR "Implementation Science*" OR "Implement*" OR "Develop*" OR "Execut*" OR "Appl*" OR "Perform*" OR "Realiz*" OR "Accomplish*" OR "Innovat*" OR "Deploy*" OR "Post-deploy*" OR "Introduc*" OR "Adopt*" OR "Integrat*" OR "Opening*" OR "Origin*" OR "Begin*" OR "Start*" OR "Arise" OR "Arose")
**#3**	TS=("Experience*" OR "Methods" OR "Method*" OR "Methodolog*" OR "Strateg*" OR "Instrument*" OR "Tool*" OR "Barrier*" OR "Facilitator*" OR "Health Services Accessibility" OR "Access*" OR "Motivation*" OR "Expectation*" OR "Incentive*" OR "Disincentive*" OR "Acceptance*" OR "Disapproval*" OR "Outcome Assessment, Health Care" OR "Assess*" OR "Outcome*" OR "Procedure*" OR "Program Evaluation" OR "Evaluat*" OR "Effectiveness" OR "Solution*" OR "Needs Assessment" OR "Health Services Needs and Demand" OR "Need*" OR "Health Services Research" OR "Process Assessment, Health Care" OR "Process" OR "Knowledge" OR "Knowledge Discovery" OR "Discover*" OR "Knowledge" OR "Management*" OR "Systems Analysis" OR "Cost-Benefit Analysis" OR "Analysis" OR "Monitoring" OR "Quality" OR "Guideline*" OR "Patient Reported Outcome Measures" OR "Health Knowledge, Attitudes, Practice" OR "Attitude" OR "Attitude*" OR "Attitude of Health Personnel" OR "Professional Practice" OR "Institutional Practice" OR "Practice Patterns, Pharmacists" OR "Evidence-Based Practice" OR "Practice*" OR "Organization and Administration" OR "Administrati*" OR "Administer*" OR "Procedures and Techniques Utilization" OR "Planning Techniques" OR "Technique*" OR "Planning" OR "Evaluation Studies as Topic" OR "Structuring" OR "Patient Care Management" OR "System*" OR "Change Management" OR "Change*" OR "Benefit*" OR "Risk" OR "Risk*" OR "Improvement*" OR "Quality Indicators, Health Care" OR "Indicator*" OR "Health Care Quality, Access, and Evaluation" OR "Quality of Health Care" OR "Management Audit" OR "Audit" OR "Guideline Adherence" OR "Adherence" OR "Expert Testimony" OR "Public Opinion" OR "Opinion*" OR "Learning" OR "Learning" OR "Education, Professional" OR "Fragility" OR "Evolution*" OR "Transformation*" OR "Strength*" OR "Weaknesse*" OR "Recommendation*" OR "Growth*" OR "Research Report" OR "Progress" OR "Instruction*" OR "Goals" OR "Goal*" OR "Organizational Objectives" OR "Objective*" OR "Advance*" OR "Conflict*" OR "Feedback" OR "Feedback*" OR "Behavior" OR "Behavior*" OR "Capacity Building" OR "Inservice Training" OR "Training*" OR "Qualification*" OR "Professional Competence" OR "Clinical Competence" OR "Competence*" OR "Aptitude" OR "Ability*" OR "Skill*" OR "News" OR "Preliminary Data" OR "Culture" OR "Culture*" OR "Background*" OR "Staff Development" OR "Acceptability")
